# Monitoring of SARS-CoV-2 Specific Antibodies after Vaccination

**DOI:** 10.3390/vaccines10020154

**Published:** 2022-01-20

**Authors:** Raquel Guiomar, Ana João Santos, Aryse Martins Melo, Inês Costa, Rita Matos, Ana Paula Rodrigues, Irina Kislaya, Anabela Santos Silva, Carla Roque, Carla Nunes, Joaquim Aguiar, Fátima Graça, Antônio Silva Graça, Ausenda Machado

**Affiliations:** 1 National Reference Laboratory for Influenza and Other Respiratory Viruses, Infectious Diseases Department, National Institute of Health Doutor Ricardo Jorge, 1649-016 Lisbon, Portugal; raquel.guiomar@insa.min-saude.pt (R.G.); ines.costa@insa.min-saude.pt (I.C.); 2 Epidemiology Department, National Institute of Health Doutor Ricardo Jorge, 1649-016 Lisbon, Portugal; ana.carvalho@insa.min-saude.pt (A.J.S.); ana.rodrigues@insa.min-saude.pt (A.P.R.); irina.kislaya@insa.min-saude.pt (I.K.); ausenda.machado@insa.min-saude.pt (A.M.); 3 Immunology Laboratory for Infectious Diseases, Infectious Diseases Department, National Institute of Health Doutor Ricardo Jorge, 1649-016 Lisbon, Portugal; rita.matos@insa.min-saude.pt; 4 COVID-19 Working Group, National Institute of Health Doutor Ricardo Jorge, 4000-055 Porto, Portugal; anabela.silva@insa.min-saude.pt (A.S.S.); carla.nunes@insa.min-saude.pt (C.N.); joaquim.aguiar@insa.min-saude.pt (J.A.); m.fatima.graca@insa.min-saude.pt (F.G.); 5 Occupational Health Department, National Institute of Health Doutor Ricardo Jorge, 1649-016 Lisbon, Portugal; carla.roque@insa.min-saude.pt (C.R.); silva.graca@insa.min-saude.pt (A.S.G.)

**Keywords:** COVID-19, immunology, health care workers, neutralizing antibodies

## Abstract

Vaccination is considered the most important measure to control the COVID-19 pandemic. Extensive follow-up studies with distinct vaccines and populations are able to promote robust and reliable data to better understand the effectiveness of this pharmacologic strategy. In this sense, we present data regarding binding and neutralizing (achieved by surrogate ELISA assay) antibodies throughout time, from vaccinated and previously infected (PI) health care workers (HCW) in Portugal. We analyzed serum samples of 132 HCW, who were vaccinated and with previous SARS-CoV-2 infection. Samples were collected before vaccination (baseline, M1), at second dose vaccine uptake (M2), and 25–70 days (M3) and 150–210 days (M4) after the second dose for vaccinated individuals. The IgG (anti-RBD/S) antibody geometric mean titers found on vaccinated HCW at M2 (GM = 116.1 BAU/mL; CI: 92.3–146.1) were significantly higher than those found on PI HCW at recruitment (M1) (GM = 35.9 BAU/mL; CI:15.4–83.4), and the neutralizing antibodies (nAb) were similar between these groups, of 93.2 UI/mL (95% CI 73.2–118.5) vs. 84.1 UI/mL (95% CI 40.4–155.9), respectively. We detected around 10-fold higher IgG (anti-RBD/S) antibodies titers in M3 when compared with M2, with a slight but significant decrease in titers from 36 days after the second dose vaccine uptake. The increase of nAb titers was correlated with IgG (anti-RBD/S) antibodies titers; however, in contrast to IgG (anti-RBD/S) antibodies titers, we did not detect a decrease in the nAb titer 36 days after a second vaccine dose uptake. At M4, a decrease of 8-fold in binding IgG (anti-RBD/S) and nAb was observed. No significant differences in antibody titers were observed by sex, age or chronic diseases. Our results suggest that IgG (anti-RBD/S) antibodies titers and nAb titers could be correlated, but an ongoing follow up of the cohort is required to better understand this correlation, and the duration of the immune response.

## 1. Introduction

Vaccination is an important public health measure to control the Coronavirus Disease 2019 (COVID-19) caused by the Severe Acute Respiratory Syndrome Coronavirus 2 (SARS-CoV-2). Substantial efforts have been made worldwide to develop vaccines that are able to protect against COVID-19 [1]. Multiple mechanisms were used in vaccines development, among them, the novel technology of mRNA-based vaccines [2], such as Comirnaty^®^ and Spikevax^®^ and the adenoviral vector vaccines as Vaxzevria^®^, and COVID-19 Vaccine Janssen^®^ which are being used in the vaccination program of Portugal, to date [3].

In the actual pandemic context, given the record time from the research to the production and mass application of vaccines, the follow-up of vaccinated people is essential to obtain data regarding antibody response among different populations, under different epidemiological contexts [4]. In addition to the vaccine-effectiveness estimates, studies on immunogenicity are important to monitor vaccine performance.

The Spike protein (S) of SARS-CoV-2 is actually the primary target used in vaccine development, since the receptor-binding domain (RBD), in subunit S1 of this protein, is considered the main target to binding and neutralizing antibodies [5]. Thus, it is expected that vaccinated people present anti-protein S antibodies, while people with previous COVID-19 may present anti-protein S and anti-nucleoprotein antibodies [6].

Previous studies demonstrated high seroconversion (up to 90%) in both vaccinated and infected people, although high heterogeneity among individuals was observed [7,8,9]; however, the duration of the adaptive immune response has not yet been well established. Some studies have demonstrated antibodies persistence in previously infected individuals for, at least, 12 months after symptoms onset [7], and at least 6 months after the complete vaccination scheme in vaccinated individuals [10].

The correlation between binding and neutralizing antibodies titers and protection is not clear in infection by SARS-CoV-2, and a cut off to predict protection is not available. A meta-analysis study based on seven different COVID-19 vaccines evidenced a correlation between binding and neutralizing antibodies with protection against symptomatic COVID-19, indicating the use of antibodies tests to correlate protection against disease, but no threshold was established [11].

In order to clarify questions regarding then heterogeneity of immune response among individuals, duration of the adaptive immune response, correlation among biding and neutralizing antibodies and protection, the future need of vaccines boosters, among other factors, it is essential to conduct studies based on real-life observation of different populations and of different vaccines. This is particularly true for studies in which the serological response and vaccine effectiveness measured at the same time. The answers to these questions are an important key to delineate the next steps to control the COVID-19 pandemic.

In Portugal, the vaccination program against COVID-19 began in 27 December 2020. As in the majority of European countries, the National Directorate of Health and the Ministry of Health defined a strategy that prioritized front-line Health Care Workers (HCW) for vaccination, since they are essential to maintain the health care units operational during the pandemic, but also because they are at higher risk of SARS-CoV-2 infection, and given the close contact between these professionals and patients with comorbidities, with an increased possibility of virus transmission to patients with high risk for severe disease [12]. The first vaccines to become available in Portugal were the Comirnaty^®^ vaccine followed by Spikevax^®^ (both vaccines recommended to individuals ≥ 12 years of age); Vaxzevria^®^ (first recommended to individuals aged less than 65 years and then to individuals aged 60 or more years) and the Janssen^®^ COVID-19 Vaccine (recommended to women aged ≥50 years old or adults men) [3]. Following the national vaccination guidelines, during the study period, those people with previous infection were vaccinated after 6 months from laboratory diagnosis, with one dose of any of the vaccines [13].

The National Institute of Health Dr. Ricardo Jorge (INSA) has approximately 500 HCW in its staff, within which 81 were considered as front-line HCW to receive the vaccine against COVID-19 in the first phase of the national vaccination program.

Assuming the importance of specific SARS-CoV-2 antibodies as a proxy of the immune response to COVID-19 vaccines, HCW of INSA were followed-up for the first 6 months after the second vaccine-dose uptake. In this study, we report findings regarding binding and neutralizing antibodies in vaccinated and previously infected HCW of INSA, in Portugal, from the initial moment (recruitment or the first vaccine dose uptake) to 6 months after the second vaccine-dose uptake.

## 2. Materials and Methods

### 2.1. Design and Participants

A prospective cohort study among INSA’s staff was implemented to examine SARS-CoV-2 vaccine effectiveness, including the serological component.

The INSA institutional vaccination campaign began on 12 January 2021. HCW were invited to participate and were assigned to either the vaccinated cohort or the non-vaccinated cohort at the beginning of the follow-up period. Individuals were asked to participate via institutional email by the occupational medical service.

Initially, all participants filled-in a recruitment questionnaire in which risk factors, symptoms and vaccination data were collected. In addition, all participants were followed on a weekly basis, by fill-in an online questionnaire on SARS-CoV-2 exposure and infection symptoms. Nasopharyngeal/oropharyngeal swab was collected for SARS-CoV-2 detection by RT-PCR tests when participants reported suspected signs and symptoms of COVID-19 on the weekly questionnaire or under the periodic testing screening at INSA.

### 2.2. Inclusion Criteria

All staff of INSA that consented to participate in our cohort study of vaccine effectiveness against COVID-19 were included in this research project.

### 2.3. Exclusion Criteria

The participants of our cohort study who did not received any dose of vaccine or that had not experienced a previous infection were excluded from this analysis.

### 2.4. Definitions

We considered as vaccinated, individuals for whom 14 days had elapsed following complete vaccination (receiving all doses recommended in the product characteristics); and individuals as partially vaccinated, if 14 days had elapsed after receiving the first dose and until 14 days after receiving the second dose (in case of 2 doses). Additionally, individuals were classified in the “previous infection” group if they reported a positive RT-PCR test to SARS-CoV-2 or had a positive RT-PCR test to SARS-CoV-2 and/or had anti-SARS-CoV-2 antibodies in a serum sample taken in the recruitment at Moment 1(M1), before vaccine uptake.

### 2.5. Serological Tests

Blood samples (3–5 mL) were collected through venipuncture at the beginning (M1) for all participants (including previously infected individuals), 30 days after their first vaccine-dose uptake (M2), 30 days after second dose (M3) and at 6 months follow up (M4). After centrifugation at 2500 rpm for 15 min, serum was obtained and conserved refrigerated (2–8 °C) for a maximum of 7 days before laboratorial analysis to detection of IgG antibodies and then, were stored frozen (−20 °C) until the neutralizing antibodies test was performed.

### 2.6. Determination of IgG Antibodies

The determination of SARS-CoV-2 specific antibodies was performed by a chemiluminescence enzyme immunoassay used for the quantitative detection of Anti- receptor-binding domain (RBD) from spike protein (S) antibodies (IgG anti-RBD/S) against SARS-CoV-2. Assays were performed in serum samples by the SARS-CoV-2 IgG II Quant assay (Abbott Diagnostics, Abbott Park, IL, USA). Sera samples were considered positive when they presented results of >50 AU/mL (>7.1 BAU/mL). The tests were performed according to the manufacturer’s instructions.

The determination of IgG (anti-RBD/S) was performed at baseline, before the first vaccine dose uptake (M1), before second vaccine dose uptake (M2), 25 to 70 days after second vaccine dose or completion of vaccination scheme (M3), and at 150 and 210 days after second vaccine dose or completion of vaccination scheme (M4).

### 2.7. Determination of Neutralizing Antibodies

The determination of neutralizing antibodies was performed at M2, M3 and M4 for vaccinated participants and at M1 for participants that had a previous infection, using the commercial surrogate Enzyme-Linked Immunosorbent Assay (ELISA) kit TECO^®^ SARS-CoV-2 Neutralization Antibody Assay (TECOmedical AG, Sissach, Switzerland), in the fully automated ELISA System DYNEX DS2^®^ (Chantilly, VA, USA). The test has the principle of competitive binding, based on protein–protein interaction from the virus spike (S) protein (receptor binding domain—RBD) and the host cell receptor protein (angiotensin-converting enzyme 2-ACE2), and, according to the manufacturer, presents 99% sensitivity and 100% specificity when compared with the gold standard plaque reduction neutralization test (PRNT). The test was performed according to the manufacturer’s instructions. The interpretation of the results was performed by the DYNEX DS2^®^ system, and the results in IU/mL (calculated based on the standard 20/136 of NIBSC/WHO) were obtained for the optic density (OD) of each sample. The limit of interpretation of the equipment is in the interval between 5 IU/mL and 500 IU/mL. Those samples that presented a value >500 IU/mL to nAb were re-analyzed in dilutions of 5× or 10×, to obtain the most robust result possible to perform the statistical correlation among binding and neutralizing antibodies. The cutoff to determine the presence of neutralizing antibodies was established as ≥20 IU/mL.

### 2.8. Statistical Analyses

Demographic, social and health characteristics of vaccinated (including partially vaccinated) individuals at baseline are described as relative frequencies for categorical, mean and standard deviations for numerical variables.

For vaccinated, partially vaccinated and individuals with previous infection of COVID-19, data on the quantification of IgG (anti-RBD/S) and nAb antibody response activity are represented as an estimated geometric mean (GM) with a 95% confidence interval (95% CI). The Wilcoxon’s rank sum test was applied to detect statistical differences for the IgG (anti-RBD/S) titers (GMT) at different moments. Between the 1st dose (20–30 days after vaccination, M2) and 2nd vaccine dose or completion of vaccination scheme (25–70 days after vaccination, M3), and between these two moments and 5 to 7 months after vaccination (150 to 210 days after 2nd dose or 1st dose in one uptake vaccine scheme, M4). Given the difference of the elapsed time since their completion of the vaccination scheme and blood sample collection at the M3, we also tested the differenced in GMT for two points after vaccination-scheme completion (25–35 days vs. 36–70 days). Spearman’s coefficient and p-value were calculated to evaluate the correlation between IgG (anti-RBD/S) and neutralizing response activity (nAb). A linear regression was performed on log-transformed IgG (anti-RBD/S) titers at the M3 (25 to 70 days after 2nd vaccine dose) and at the M4 (150 to 210 days after 2nd vaccine dose) to determine the association to sex, age groups (20–39, 40–70 years) or chronic disease (at least one chronic disease/no disease) for vaccinated individuals. Statistical significance was defined as *p* < 0.05.

All statistical analyses were performed using Stata software, version 15.1 (StataCorp.2017. Stata Statistical Software, StataCorp, College Station, TX, USA).

### 2.9. Ethical Considerations

The study complied with the relevant legal and ethical requirements. The study protocol was approved by the National Institute of Health Doctor Ricardo Jorge Health Ethics Committee. All participants provided their written informed consent for collection of data regarding demographic, social, and health information, blood samples and nasopharyngeal/oropharyngeal swabs.

## 3. Results

Out of the 212 workers that were part of the INSA cohort on 15 June 2021, 132 were included in this study, and 114 were from the vaccinated and partially vaccinated group and 18 individuals were from the group with previous infection, and had an RT-PCR positive test (*n* = 14) or with a positive serological analysis with the detection of antibodies against SARS-CoV-2 in serum sample (*n* = 4). These four individuals were therefore unaware of having been exposed to the SARS-CoV-2.

From the vaccinated group, 84 were vaccinated against SARS-CoV-2, and 30 were partially vaccinated (Table 1). Vaccinated and partially vaccinated individuals had mostly taken the Comirnaty^®^ vaccine (88.6%, *n* = 101), followed by the Vaxzevria^®^ (6.1%, *n* = 7), the Spikevax (3.5%, *n* = 4), and the others had COVID-19 Vaccine Janssen^®^ (1.9%, *n* = 2). In both groups, the majority were women (81% and 90%, respectively), and the mean age was lower for the vaccinated group (x^−^ = 44 (23–67) versus x^−^ = 57 (46–69)). Within the vaccinated group, over 68% reported working in the laboratory, whereas over 81% of the partially vaccinated group reported working in services that did not required interaction with the public. Regarding smoking habits, 11% of the fully vaccinated individuals and 23% of the partially vaccinated were reported to be smokers. Just under half of the vaccinated (48.7%) and 68.4% of the partially vaccinated participants reported at least one chronic disease. In both groups, over 70% of participants reported to have uptake the influenza vaccine in the previous season.

**Figure 1:**
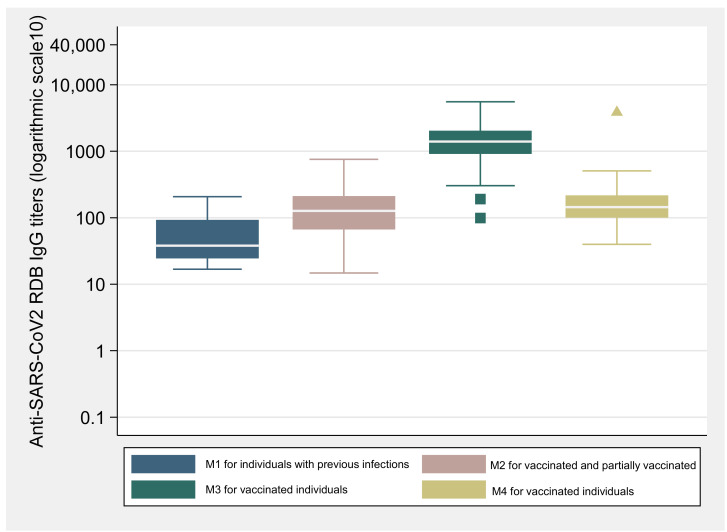
Concentration of IgG anti-SARS-CoV-2 spike receptor-binding domain (RBD) titers (logarithmic scale 10) reported in the box-whisker plots (and outliers) for individuals with previous infection at M1 and vaccinated individuals without previous infection for the three different moments of observation.

At M1, the IgG (anti-RBD/S) titers were lower for individuals with previous SARS-CoV-2 infection, compared to individuals vaccinated with one dose at M2 (Figure 1). Information about the date of the positive PCR test was only available for 11 of the 18 individuals with previous infection. Individuals that have had previous infection in the last 90 days presented a higher GM (GM = 12.7BAU/mL; CI: 3.4–47.2) than those individuals that had been infected prior to 90 days (GM = 8.2 BAU/mL; CI: 3.7–18.2).

Table 2 displays the GM for IgG (anti-RBD/S) titers for different groups and moments. The concentration of IgG (anti-RBD/S) was significantly higher (GM = 116.1 BAU/mL; CI: 92.3–146.1) for the vaccinated individuals at M2, when compared to the values observed at M1 for the individuals previously infected (GM = 35.9 BAU/mL; CI:15.4–83.4) (*p* < 0.001).

For vaccinated participants, antibody titers were significantly higher after the second vaccine dose, with an increase of around 10-fold from M2 to M3 in the IgG (anti-RBD/S) antibodies titers (GM = 1250.1 BAU/mL, 95% CI: 1069.2–1461.7) (*p* < 0.0001). For the M3, serum samples were drawn at several distinct times, comprising a broad range of days between sample collection and second dose uptake. Vaccinated individuals whose blood samples were drawn between 25 and 35 days after the second dose presented a higher GM of IgG (anti-RBD/S) concentration (GM = 1478.1 BAU/mL, CI: 1208.1–1808.3), compared to those whose sample collection took place after the 35 until 70 days (GM = 967.3 BAU/mL, CI: 766.9–1220.2) (*p* < 0.05). Even though the concentration of IgG (anti-RBD/S) titers decreased at M4 (GM = 152.0 BAU/mL; CI: 131.0–176.4) and was significantly lower when compared to the values observed at M3 (*p* < 0.001). Antibody titers at M4 were still significantly higher than observed for the vaccinated individuals at M2, after the 1st vaccine dose (*p* < 0.05). Data regarding just individuals with the Comirnaty® vaccine (majority of the cohort) can be accessed in the Supplementary Materials. 

Linear regression was performed on log-transformed IgG (anti-RBD/S) titers at the M3 and M4 to determine associations with sex, age groups or chronic disease for vaccinated individuals (Table 3). No statistical differences (*p* > 0.05) in the GM of IgG (anti-RBD/S) concentration were observed between men or women, between individuals in the two age groups or between individuals without or with at least one chronic disease at each of the observed moments.

**Figure 2:**
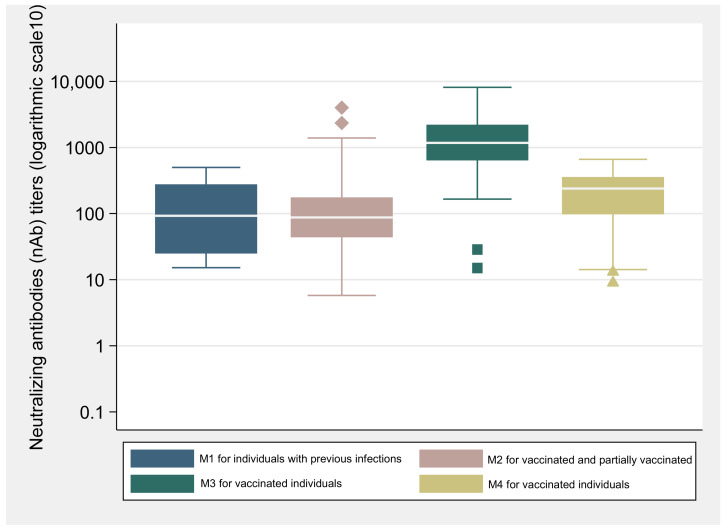
Concentration of neutralizing antibodies (nAb) titers (logarithmic scale 10) reported in the box-whisker plots (and outliers) for individuals with previous infection and vaccinated individuals without previous infection for the four different moments of observation.

To explore the humoral immune response generated after SARS-CoV-2 infection, the neutralizing antibody response (nAb) was evaluated for individuals with previous infection at recruitment (M1), and for vaccinated individuals at M2, M3 and M4 (Figure 2).

Table 4 presents the geometric mean of nAb activity in the serum samples of participants with previous infection at M1, and for vaccinated and partially vaccinated individuals at M2 and M3. The GM of nAb in previously infected individuals at M1 (GM = 84.1 mL; CI: 40.4–155.9) was not different from that observed for vaccinated individuals at M2 (GM = 93.2 IU/mL; CI: 73.2–118.5) (*p* = 0.645). On the other hand, when compared with M2, a significant increase of the nAb titer was observed in the M3 for vaccinated individuals (GM = 1267.3 IU/mL; CI: 1060.6–1514.4) (*p* < 0.001). Statistically significant differences were also observed in the nAb concentrations between 25 to 35 days (GM = 1551.9 IU/mL; CI: 1261.4–1909.5) and 36 and 70 days (GM = 935.2 IU/mL; CI: 691.6–1264.7) after the second vaccine dose or following the completion of the vaccination scheme (*p* < 0.05). Even though a decrease of nAb concentrations titers was observed in the last observation (M4, GM = 165.8 IU/mL; CI: 128.4–214.2), the titers were still significantly higher than those observed after the first vaccine dose (*p* < 0.05).

Highly statistically significant correlations were observed between IgG (anti-RBD/S) and nAb titers, at all observational points. After the first vaccine dose (M2) IgG (anti-RBD/S) titers correlated highly with nAb titers (Spearman’s ρ = 0.79, *p* < 0.001). Similar higher correlations were observed at M3 (Spearman’s ρ = 0.86, *p* < 0.001), from 25 to 35 days (Spearman’s ρ = 0. 81, *p* < 0.001) or 36 to 70 days after completion of vaccination scheme (Spearman’s ρ = 0. 87, *p* < 0.001). Finally, at M4, a significant correlation, was observed between IgG (anti-RBD/S) and nAb titers (Spearman’s ρ = 0.70, *p* < 0.001).

## 4. Discussion

In this work, we accessed preliminary data regarding binding and neutralizing antibodies in HCW from the National Public Health Institute, Portugal, from the baseline, before first vaccine dose uptake, until 150–210 days (5–7 months) after the second vaccine dose. We demonstrated that the first vaccine dose elicited an immunological response although a second dose was essential to promote binding and neutralizing antibodies SARS-CoV-2. IgG (anti-RBD/S) were highly correlated with neutralizing antibodies, being higher in the first 70 days (10 weeks), keeping however a good correlation after completion of the vaccination scheme.

The IgG (anti-RBD/S) titer was significantly higher in vaccinated individuals, after one or two vaccine doses, compared with the baseline values of individuals that had developed an immunity response after a previous SARS-CoV-2 infection. The GM of IgG (anti-RBD/S), after one vaccine dose, from vaccinated individuals was significantly higher (*p* < 0.001) than GM found at the point of recruitment for individuals that had a previous infection. These data are in accordance with the results previously reported from a cohort of HCW from an academic medical center in Southern California [14] and with the Portuguese national serological survey performed in February–March 2020 [15]. However, we have to take in consideration that the elapsed time between infection and the recruitment to this study varied greatly among individuals. Due to the lower sample size between these groups, it is not possible to perform a robust comparative analysis in the group of previous SARS-CoV-2 infection.

The marked increase of the IgG (anti-RBD/S) after the second dose found in this study was compatible with that observed in previous studies [6,8,10,14], and in trials studies [16]. It is important to highlight that after the second dose, we found a significant decrease in IgG (anti-RBD/S) between individuals that had a serum analysis from 25 to 35 days and those that had a serum analysis from 36 to 70 days after the second dose. This decrease was expected given that after the second dose the immune system becomes highly stimulated, with high production of antibodies; however it was not expected that the IgG titers would be kept at the maximum for a long time, and a decrease in basal titers of memory is generally observed [17,18]. This decrease was also found in other studies [10,19]. In this sense, it is important to follow up the cohort studies in order to establish how long the IgG titers remain and to try to correlate this data with nAb, and to establish a cut off that could help to predict protection against SARS-CoV-2 infection. Other studies in health care workers showed that the efficient immunological response to COVID-19 vaccines is associated with a reduction of new COVID-19 cases among those who received two doses of the vaccine, even when a surge of the B.1.1.7 variant was noted in up to 80% of cases. The effective vaccination among health care workers provides a safe environment, even in the presence of a high rate of SARS-CoV-2 infection in the community. Although there is a good effectiveness for COVID-19 vaccines, a decrease in the vaccine’s effectiveness with time has been recognized [20]. A study conducted in England found high levels of vaccine effectiveness against symptomatic diseases after two doses, even when the new Delta variant was circulating [21]. New variants can also pose a challenge to COVID-19 vaccine effectiveness that expresses the perfusion stabilized full spike glycoprotein (S) of the original SARS-CoV-2 isolate Wuhan-Hu-1, but recent studies have already highlighted that variants of concern [22], Alpha (B.1.1.7 variant), Beta variant first identified in South Africa (B.1.351 lineage), and Gamma variant first identified in Brazil (P.1 lineage) remained susceptible to the Comirnaty^®^ vaccine, which elicited serum neutralization, although at a reduced level for the B.1.351 variant [23,24]. For the Delta variant, which has been predominant in Portugal since mid-June 2021 [25], studies on cross-reactivity of monoclonal antibodies to pre-existing SARS-CoV-2 strains, showed that a vaccination of previously infected individuals is likely to be protective against a large array of circulating viral strains, including the Delta variant. In the same way, a two dose regimen generated high sera-neutralization levels against the Alpha, Beta and Delta variants in individuals sampled at week 8 to week 16 after vaccination [26], with the levels of neutralizing antibodies being highly predictive of immune protection from symptomatic SARS-CoV-2 infection [27]. A study at the national level estimated high mRNA VE for the prevention of COVID-19-related hospitalizations and deaths (in ≥ 65 years, full vaccinated) with evidence of VE reduction in the 3 months after the second dose uptake, during the period of Delta variant circulation [28], which is consistent with the high titers for binding and neutralizing antibodies detected after complete vaccination. Now, with the emergence of the Omicron variant [29], all the questions regarding VE against VOCs have been reinforced. In Portugal, the prevalence of the Omicron variant is rapidly increasing [25], concurrently to HCW and the general population of up to 50 years old are receiving the third vaccine dose. In this sense, monitoring the immunological status of HCW in this new scenario is of extreme importance to understand the dimension of this new VOC challenge.

In contrast to other studies such as the one performed in the UK, where people older than 50 years old presented a weaker serological response than those younger than 50 years old [30], we did not identify any difference between age, sex, or the presence of one or more chronic diseases in our study. This may be due to the limited sample size of our study, which does not allow for a more robust analysis.

Our study has some limitations, as the cohort includes only active healthy workers, without severe comorbidities, and all of our participants were under 70 years old, with an over representation of the female population. The occupational risk is reduced, although the majority of the participants manipulate SARS-CoV-2 positive samples, strict guidelines to use individual protective equipment limit the occupational risk exposure being reduced compared to medical personnel with close contact with patients. The heterogeneity in the reference units to quantify the detected antibodies posed a difficulty to compare our data with other studies. Few studies used the same units that we used in this study. This point has already been highlighted by Earle et al., [11] who suggested the use of the WHO International Standard (NIBSC 20/136) to express neutralizing antibodies titers in IU/mL and binding antibodies titers in BAU/mL to compare data among different studies using serology assays, in the context of the COVID-19 pandemic. Given the importance of comparing different vaccines among different populations to help to design new strategies to battle the pandemic, this point is crucial to a better comprehension of data obtained around the world. We did not explore the antibody titers for variants of concern, and a decrease in antibody titers could be expected. The cellular immunity and other immunological mechanisms were not explored during the study.

In our study, we found a high correlation between biding and neutralizing antibodies after the first dose and similar results have been previously reported by other authors [31,32]. It was reported that nAb remains relatively stable for several months after infection, but there is a lack of information on the duration for which it persists [32]. The slower decrease in nAb after the second vaccine dose uptake could support a robust and long persistence of nAB after full vaccination [20,32,33]. A recent study reported a good correlation between binding and neutralizing antibodies levels and protection against symptomatic infection [34]. Although we observed a decrease in the level of nAb titers at around 6 months after vaccination, it was much higher than the minimum level reported by Feng et al., and was associated with 80%VE against symptomatic infection, primarily with the Alpha variant (26 IU/mL for pseudovirus neutralization). In our study, we were not able to predict the loss of protection due to the decrease in nAb 5–7 months after full vaccination, so we were unable confirm whether the nAb levels found at this moment were considered a robust immune response. In this sense, we recommend caution in the interpretation of the decrease in the levels of biding and neutralizing antibodies, especially when facing challenges presented by new variants. Regarding the methodology used to measure the nAb, we recognize that classical methods such as the plaque reduction neutralization test (PRNT) are the gold standard to measure nAb titers; however, this methodology is time-consuming and requires increased biosafety procedures, since live viruses are used in these tests. Thus, we chose to work with a surrogate ELISA assay after some previous studies had demonstrated that, although less sensitive, surrogate assays are appropriate for application in cohort studies [35,36,37,38]. The surrogate ELISA may present some false positive or false negative results, mainly to those sera in the grey zone; however, this test has a good applicability in population studies. In this study, we considered a good correlation among binding and neutralizing antibodies as observed in previous studies.

The preliminary data obtained from our cohort study demonstrate the importance to follow up with individuals in order to better understand the behavior of the body’s immune response to the COVID-19 vaccines, and to try to establish a threshold that could predict protection against the SARS-CoV-2 infection. Serological studies preclude the waning of antibody levels with time, and must be integrated in the vaccine effectiveness studies to better respond to questions about the duration of immune protection and vaccine effectiveness. The data on waning immunity and vaccine effectiveness are valuable for health decision makers, to implement measures to reduced severe disease, mortality and transmission that could include non-pharmaceutical measures and/or additional booster vaccine doses.

## Figures and Tables

**Table 1:** Vaccinated and partially vaccinated participants’ sociodemographic, work, and health characteristics.

	Vaccinated		Partially Vaccinated (1 Dose) *	
	*n*	%	*n*	%
Total	84		30	
Sex				
Female	68	81.0	27	90.0
Male	16	19.0	3	10.0
Age, mean [range]	(x^−^ = 44 [23–67])		(x^−^ = 57 [46–69])	
Age groups (*n*)	(83)		(30)	
20 to 49 years	44	53.0	5	16.7
50 to 70 years	39	47.0	25	83.3
Work functions (*n*)	(79)		(26)	
Without public contact	13	16.5	21	80.8
Public contact	12	15.2	4	15.4
Laboratory	54	68.4	1	3.8
Smoking (*n*)	(82)		(30)	
Never smoked	54	65.9	16	53.3
Smoker	9	11.0	7	23.3
Former smoker	19	23.2	7	23.3
Chronic disease (*n*)	(70)		(19)	
No disease	38	54.3	6	31.6
1+ disease	32	45.7	13	68.4
Flu vaccine season 2020/2021 (*n*)	(81)		(28)	
No	24	29.3	5	17.9
Yes	58	70.7	23	82.1

* Partially vaccinated individuals were only considered for M1 and M2.

**Table 2:** Geometric Mean (GM) of SARS-CoV-2 IgG (anti-RBD/S) concentration titers at four different moments for the full-vaccinated, partially vaccinated participants and at moment of recruitment for individuals with previous infection.

Groups	M1 [CI 95%] (*n*)	M2 (Vaccinated and Partial) [CI 95%] (*n*)	M3 (Vaccinated)			M4 (Vaccinated)
			Total [CI 95%] (*n*)	25–35 Days [CI 95%] (*n*)	36–70 Days [CI 95%] (*n*)	150–210 Days [CI 95%] (*n*)
Vaccinated & Partially vaccinated *	0.17 [0.14–0.20] (*n* = 96)	116.1 [92.3–146.1] (*n* = 63)	1250.1 [1069.2–1461.7] (*n* = 81)	1478.1 [1208.1–1808.3] (*n* = 49)	967.3 [766.9–1220.2] (*n* = 32)	152.0 [131.0–176.4] (*n* = 72)
Previous infection (*n* = 17)	35.9 [15.4–83.4]	_	_	_	_	_

* Partially vaccinated individuals were only considered for M1 and M2.

**Table 3:** Regression on log-transformed SARS-CoV-2 IgG (anti-RBD/S) titers at the M3 (25–70 days after 2nd dose) and M4(150–210 days after 2nd dose) for full-vaccinated individuals.

		M3			M4	
Variable	Odds Ratio	95% CI	*p*	Odds Ratio	95% CI	*p*
Sex	(*n* = 87)			(*n* = 72)		
Female	*			*		
Male	0.83	0.56–1.2	0.358	0.79	0.51–1.2	0.298
Age	(*n* = 86)			(*n* = 72)		
20–49	*			*		
50–70 years	1.08	0.79–1.48	0.626	0.86	0.60–1.2	0.415
Chronic disease	(*n* = 71)			(*n* = 61)		
No disease	*			*		
1 + disease	1.19	0.84–1.69	0.310	0.95	0.70–1.3	0.776

* Constant, reference category.

**Table 4:** Geometric Mean (GM) of neutralizing antibodies (nAb) titers at four different moments for the full-vaccinated, partially vaccinated participants and at moment of recruitment for individuals with previous infection.

Groups	M1 [CI 95%] (*n*)	M2 (Vaccinated and Partial) [CI 95%] (*n*)	M3 (Vaccinated)			M4 (Vaccinated)
			Total [CI 95%] (*n*)	25–35 Days [CI 95%] (*n*)	36–70 Days [CI 95%] (*n*)	150–210 Days [CI 95%] (*n*)
Vaccinated & Partially vaccinated *	_	93.2 [73.2–118.5] (*n* = 84)	1267.3 [1060.6–1514.4] (*n* = 80)	1551.9 [1261.4–1909.5] (*n* = 48)	935.2 [691.6–1264.7] (*n* = 32)	165.8 [128.4–214.2] (*n* = 68)
Previous infection (*n* = 14)	84.1 [40.4–155.9]	_	_	_	_	_

* Partially vaccinated individuals were only considered for M1 and M2.

## Data Availability

The data presented in this study are available on request from the corresponding author. The data are not publicly available due to confidential procedures stated in the Informed Consent.

## Supplementary Materials

The following supporting information can be downloaded at: https://www.mdpi.com/article/10.3390/vaccines10020154/s1, Figure S1. Concentration of IgG anti-SARS-CoV-2 spike receptor-binding domain (RBD) titers (logarithmic scale 10) reported in the box–whisker plots (and outliers) for individuals with previous infection at M1 and vaccinated with the Cominarty vaccine individuals without previous infection for the three different moments of observation. Table S1. Geometric Mean (GM) of SARS-CoV-2 IgG (anti-RBD/S) concentration titers at four different moments for participants full-vaccinated and partially vaccinated, with the Comirnaty^®^ vaccine.

## References

[1] WHO (2021). Evaluation of COVID-19 Vaccine Effectiveness.

[2] Forni G., Mantovani A., Forni G., Mantovani A., Moretta L., Rappuoli R., Rezza G., Bagnasco A., Barsacchi G., Bussolati G. (2021). COVID-19 vaccines: Where we stand and challenges ahead. Cell Death Differ..

[3] Infarmed Infarmed COVID-19 -Vacinas Aprovadas (Quadro Resumo) 2021. https://www.infarmed.pt/web/infarmed/vacinas-aprovadas.

[4] Carrillo J., Izquierdo-Useros N., Ávila-Nieto C., Pradenas E., Clotet B., Blanco J. (2021). Humoral immune responses and neutralizing antibodies against SARS-CoV-2; implications in pathogenesis and protective immunity. Biochem. Biophys. Res. Commun..

[5] Lombardi A., Bozzi G., Ungaro R., Villa S., Castelli V., Mangioni D., Muscatello A., Gori A., Bandera A. (2021). Mini Review Immunological Consequences of Immunization with COVID-19 mRNA Vaccines: Preliminary Results. Front. Immunol..

[6] Gobbi F., Buonfrate D., Moro L., Rodari P., Piubelli C., Caldrer S., Riccetti S., Sinigaglia A., Barzon L. (2021). Antibody Response to the BNT162b2 mRNA COVID-19 Vaccine in Subjects with Prior SARS-CoV-2 Infection. Viruses.

[7] Dobaño C., Ramírez-Morros A., Alonso S., Vidal-Alaball J., Ruiz-Olalla G., Vidal M., Rubio R., Cascant E., Parras D., Melero N.R. (2021). Persistence and baseline determinants of seropositivity and reinfection rates in health care workers up to 12.5 months after COVID-19. BMC Med..

[8] Callegaro A., Borleri D., Farina C., Napolitano G., Valenti D., Rizzi M., Maggiolo F. (2021). Antibody response to SARS-CoV-2 vaccination is extremely vivacious in subjects with previous SARS-CoV-2 infection. J. Med. Virol..

[9] Manisty C., Otter A.D., Treibel T.A., McKnight Á., Altmann D.M., Brooks T., Noursadeghi M., Boyton R.J., Semper A., Moon J.C. (2021). Antibody response to first BNT162b2 dose in previously SARS-CoV-2-infected individuals. Lancet.

[10] Doria-Rose N., Suthar M.S., Makowski M., O’Connell S., McDermott A.B., Flach B., Ledgerwood J.E., Mascola J.R., Graham B.S., Lin B.C. (2021). Antibody Persistence through 6 Months after the Second Dose of mRNA-1273 Vaccine for COVID-19. N. Engl. J. Med..

[11] Earle K.A., Ambrosino D.M., Fiore-Gartland A., Goldblatt D., Gilbert P.B., Siber G.R., Dull P., Plotkin S.A. (2021). Evidence for antibody as a protective correlate for COVID-19 vaccines. Vaccine.

[12] Rudberg A.-S., Havervall S., Månberg A., Falk A.J., Aguilera K., Ng H., Gabrielsson L., Salomonsson A.-C., Hanke L., Murrell B. (2020). SARS-CoV-2 exposure, symptoms and seroprevalence in healthcare workers in Sweden. Nat. Commun..

[13] Direção-Geral da Saúde Norma No. 002/2021 de 30/01/2021 atualizada a 21/04/2021. https://backoffice.ump.pt/files/files/norma%20002.pdf.

[14] Ebinger J.E., Fert-Bober J., Printsev I., Wu M., Sun N., Prostko J.C., Frias E.C., Stewart J.L., Van Eyk J.E., Braun J.G. (2021). Antibody responses to the BNT162b2 mRNA vaccine in individuals previously infected with SARS-CoV-2. Nat. Med..

[15] Rodrigues A.P., Garcia A.C. (2021). Inquérito Serológico Nacional Relatório de Apresentação.

[16] Walsh E.E., Frenck R.W., Falsey A.R., Kitchin N., Absalon J., Gurtman A., Lockhart S., Neuzil K., Mulligan M.J., Bailey R. (2020). Safety and Immunogenicity of Two RNA-Based COVID-19 Vaccine Candidates. N. Engl. J. Med..

[17] Sethuraman N., Jeremiah S.S., Ryo A. (2020). Interpreting Diagnostic Tests for SARS-CoV-2. JAMA.

[18] Azkur A.K., Akdis M., Azkur D., Sokolowska M., Van De Veen W., Brüggen M.-C., O’Mahony L., Gao Y., Nadeau K., Akdis C.A. (2020). Immune response to SARS-CoV-2 and mechanisms of immunopathological changes in COVID-19. Allergy.

[19] Wisnewski A.V., Campillo Luna J., Redlich C.A. (2021). Human IgG and IgA responses to COVID-19 mRNA vaccines. PLoS ONE.

[20] Pilishvili T., Gierke R., Fleming-Dutra K.E., Farrar J.L., Mohr N.M., Talan D.A., Krishnadasan A., Harland K.K., Smithline H.A., Hou P.C. (2021). Effectiveness of mRNA Covid-19 Vaccine among U.S. Health Care Personnel. N. Engl. J. Med..

[21] Lopez Bernal J., Andrews N., Gower C., Gallagher E., Simmons R., Thelwall S., Stowe J., Tessier E., Groves N., Dabrera G. (2021). Effectiveness of COVID-19 Vaccines against the B.1.617.2 (Delta) Variant. N. Engl. J. Med..

[22] ECDC (2021). SARS-CoV-2 Variants of Concern as of 23 September 2021.

[23] Liu Y., Liu J., Xia H., Zhang X., Fontes-Garfias C.R., Swanson K.A., Swanson K.A., Cai H., Sarkar R., Chen W. (2020). Neutralizing Activity of BNT162b2-Elicited Serum. N. Engl. J. Med..

[24] Liu Y., Liu J., Xia H., Zhang X., Zou J., Fontes-Garfias C.R., Weaver S.C., Swanson K.A., Cai H., Sarkar R. (2021). BNT162b2-Elicited Neutralization against New SARS-CoV-2 Spike Variants. N. Engl. J. Med..

[25] Instituto Nacional de Saúde Doutor Ricardo Jorge (2021). Diversidade Genética do Novo Coronavírus SARS-CoV-2 (COVID-19) em Portugal.

[26] Planas D., Veyer D., Baidaliuk A., Staropoli I., Guivel-Benhassine F., Rajah M.M., Planchais C., Porrot F., Robillard N., Puech J. (2021). Reduced sensitivity of SARS-CoV-2 variant Delta to antibody neutralization. Nature.

[27] Khoury D.S.S., Cromer D., Reynaldi A., Schlub T.E.E., Wheatley A.K.K., Juno J.A.A., Subbarao K., Kent S.J.J., Triccas J.A.A., Davenport M.P.P. (2021). Neutralizing antibody levels are highly predictive of immune protection from symptomatic SARS-CoV-2 infection. Nat. Med..

[28] Nunes B., Rodrigues A.P., Kislaya I., Cruz C., Peralta-Santos A., Lima J., Leite P.P., Sequeira D., Dias C.M., Machado A. (2021). mRNA vaccine effectiveness against COVID-19-related hospitalisations and deaths in older adults: A cohort study based on data linkage of national health registries in Portugal, February to August 2021. Eurosurveillance.

[29] World Health Organization Update on Omicron. https://www.who.int/news/item/28-11-2021-update-on.

[30] Maria Prendecki C.C., Jonathan Brown A.C., Sarah Gleeson M.G., Paul Randell A.D.P., Liz Lightstone X.-N.X., Wendy Barclay S.P.M., Peter Kelleher M.W., Prendecki M., Clarke C., Brown J. (2021). Effect of previous SARS-CoV-2 infection on humoral and T-cell responses to single-dose BNT162b2 vaccine. Lancet.

[31] Demonbreun A.R., Sancilio A., Velez M.P., Ryan D.T., Saber R., Vaught L.A., Reiser N.L., Hsieh R.R., D’Aquila R.T., Mustanski B. (2021). Comparison of IgG and neutralizing antibody responses after one or two doses of COVID-19 mRNA vaccine in previously infected and uninfected individuals. EClinicalmedicine.

[32] Wajnberg A., Amanat F., Firpo A., Altman D.R., Bailey M.J., Mansour M., McMahon M., Meade P., Mendu D.R., Muellers K. (2020). Robust neutralizing antibodies to SARS-CoV-2 infection persist for months. Science.

[33] Saad-roy C.M., Wagner C.E., Baker R.E., Morris S.E., Farrar J., Graham A.L., Levin S.A., Mina M.J., Metcalf C.J.E., Grenfell B.T. (2020). Immune life history, vaccination, and the dynamics of SARS-CoV-2 over the next 5 years. Science.

[34] Feng S., Phillips D.J., White T., Sayal H., Aley P.K., Bibi S., Dold C., Fuskova M., Gilbert S.C., Hirsch I. (2021). Correlates of protection against symptomatic and asymptomatic SARS-CoV-2 infection. Nat. Med..

[35] Chan K.-H., Leung K.-Y., Zhang R.-R., Liu D., Fan Y., Chen H., Yuen K.-Y., Hung I.F.-N. (2021). Performance of a Surrogate SARS-CoV-2-Neutralizing Antibody Assay in Natural Infection and Vaccination Samples. Diagnostics.

[36] Kohmer N., Rühl C., Ciesek S., Rabenau H. (2021). Utility of Different Surrogate Enzyme-Linked Immunosorbent Assays (sELISAs) for Detection of SARS-CoV-2 Neutralizing Antibodies. J. Clin. Med..

[37] Tan C.W., Chia W.N., Qin X., Liu P., Chen M.I.-C., Tiu C., Hu Z., Chen V.C.-W., Young B.E., Sia W.R. (2020). A SARS-CoV-2 surrogate virus neutralization test based on antibody-mediated blockage of ACE2–spike protein–protein interaction. Nat. Biotechnol..

[38] Meyer B., Reimerink J., Torriani G., Brouwer F., Godeke G.-J., Yerly S., Hoogerwerf M., Vuilleumier N., Kaiser L., Eckerle I. (2020). Validation and clinical evaluation of a SARS-CoV-2 surrogate virus neutralisation test (sVNT). Emerg. Microbes Infect..

