# Supplementary material for: Monitoring of SARS-CoV-2 Specific Antibodies after Vaccination
Source: Vaccines (Basel). 2022 Jan 20;10(2):154. doi: 10.3390/vaccines10020154 (PMC8880181; doi:10.3390/vaccines10020154)
Supplement: Supplementary file 1 [file vaccines-10-00154-s001.zip › vaccines-1545226-supplementary.pdf]

Supplementary material

Figure S1 and Table S1 show the serology results to vaccinated and partially vaccinated individuals with the Comirnaty® vaccine (88.6%, n=101). Since the majority of the participants had taken the Comirnaty® vaccine, the results of this analysis didn't differ significantly from those reported in the main manuscript.

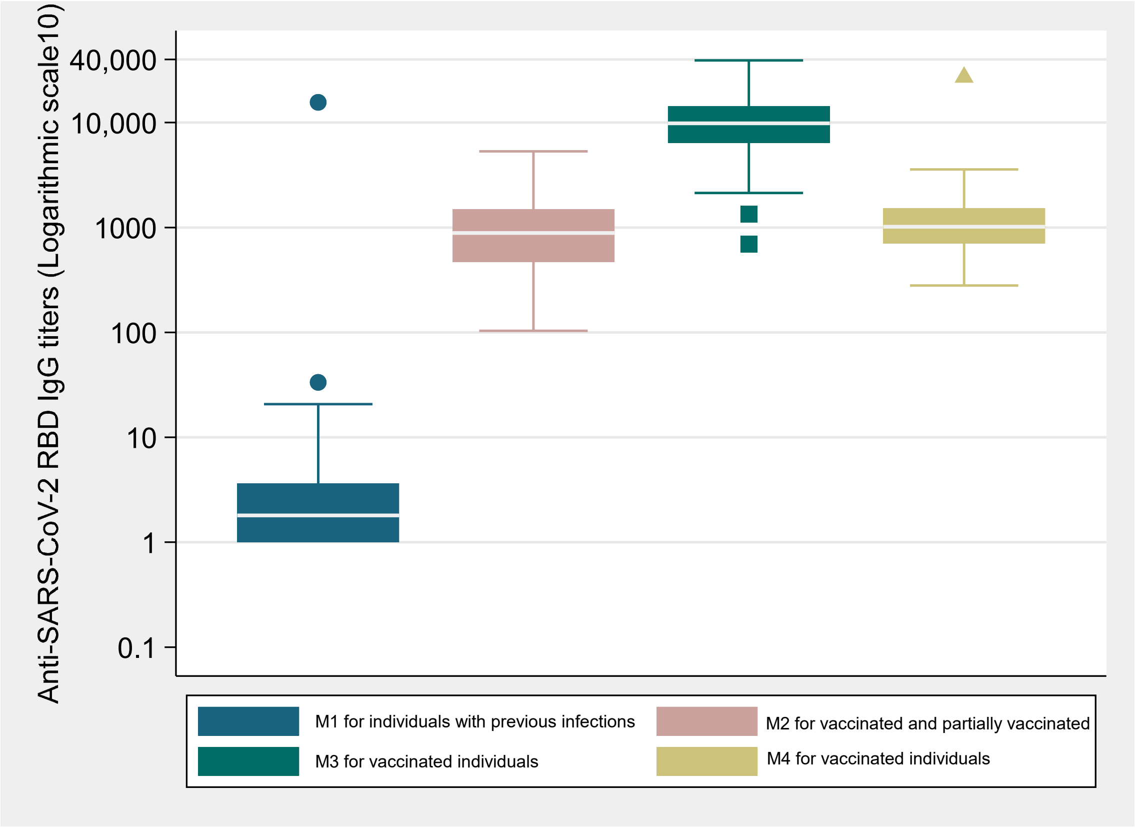

Figure S1. Concentration of IgG anti-SARS-CoV-2 spike receptor-binding domain (RBD) titers (logarithmic scale 10) reported in the box–whisker plots (and outliers) for individuals with previous infection at M1 and vaccinated with the Cominarty vaccine individuals without previous infection for the three different moments of observation.

Table S1. Geometric Mean (GM) of SARS-CoV-2 IgG (anti-RBD/S) concentration titers at four different moments for participants full-vaccinated and partially vaccinated, with the Comirnaty® vaccine

Groups	M1 [CI95%] (*n*)	M2 (Vaccinated and partial) [CI95%] (*n*)	M3 (Vaccinated)			M4 (Vaccinated)
			Total [CI95%] (*n*)	25-35 days [CI95%] (*n*)	36-70 days [CI95%] (*n*)	150-210 days [CI95%] (*n*)

Vaccinated & Partially vaccinated*	0.17 [0.14-0.21] (n=80)	116.0 [91.8-146.5] (n=62)	1250.1[1069.2-1461.7 (n=81)	1478.1[1208.1-1808.3] (n=49)	967.3 [766.9-1220.2] (n=32)	152.0 [131.0-176.4] (n=72)
